# Caractérisation des blessures sur une saison sportive chez des footballeurs d'élite au Burkina Faso: cas de la saison 2019-2020

**DOI:** 10.11604/pamj.2024.48.33.39254

**Published:** 2024-05-30

**Authors:** Adama Tiama, Alain Traoré, Abdoul Rahamane Cissé, André Kaboré, Amidou Sawadogo, Brigitte Nana, Bertin Koné, Zakaridja Soré

**Affiliations:** 1 Institut des Sciences du Sport et du Développement Humain, Université Joseph Ki-Zerbo, Ouagadougou, Burkina Faso,; 2 Commission Médicale, Fédération Burkinabè de Football, Ouagadougou, Burkina Faso,; 3 Centre Hospitalier Universitaire de Tengandogo, Université Joseph Ki-Zerbo, Ouagadougou, Burkina Faso,; 4 Centre National Médico-Sportif, Ministère des Sports et des Loisirs, Ouagadougou, Burkina Faso

**Keywords:** Footballeurs, championnat de première division, blessures, Burkina Faso, Football players, first division championship, injuries, Burkina Faso

## Abstract

**Introduction:**

les blessures représentent un évènement indésirable majeur dans la carrière du sportif et la prévention est tributaire de leurs caractéristiques. Le but de cette étude était de déterminer les caractéristiques des blessures subies par des footballeurs d'élite au Burkina Faso au cours de la saison sportive 2019-2020.

**Méthodes:**

il s'agissait d'une étude transversale, réalisée avec 160 joueurs, soumis à un questionnaire. Les tests de proportion et de χ^2^ ont été utilisés respectivement pour calculer les fréquences et déterminer les facteurs associés.

**Résultats:**

au total, 157 blessures ont été enregistrées chez 143 joueurs (89,4%). Les lésions musculaires (45,85%) et l'entorse (30,6%) étaient courantes. La majorité des blessures (52%) étaient de gravité moyenne. La cuisse (30%), la cheville (26,5%) et le genou (18%) étaient les zones les plus touchées. La fréquence des blessures était plus élevée après un contact que sans contact physique avec un autre joueur (69,5% versus 30,5; p = 0,009), lors de matchs qu'à l'entraînement (82% versus 28%; p = 0.003) et durant la phase retour que la phase allée du championnat (58,5% versus 41,5%; p= 0.02).

**Conclusion:**

la fréquence des blessures subies par des footballeurs au cours de la saison 2019-2020 était élevée. Ces blessures étaient en majorité de type musculaire et tendineux, puis affectaient davantage les membres inférieurs. Elles étaient associées aux contacts physiques, aux matchs et à la phase retour du championnat. Les mesures préventives doivent tenir compte de ces caractéristiques pour être efficace.

## Abstract (en)

**Introduction:**

injuries are a major undesirable event in sportsmen's career and prevention depends on their characteristics. The aim of this study was to determine the characteristics of injuries suffered by elite footballers in Burkina Faso during the 2019-2020 sports season.

**Methods:**

we conducted a cross-sectional study among 160 players who were administered a questionnaire. Proportion and χ^2^ tests were used to calculate frequencies and determine associated factors, respectively.

**Results:**

a total of 157 injuries were recorded in 143 players (89.4%). Muscle injuries (45.85%) and sprains (30.6%) were common. The majority of injuries (52%) were of medium severity. The thigh (30%), ankle (26.5%) and knee (18%) were the most affected areas. The frequency of injuries was higher after physical contact than without physical contact with another player (69.5% versus 30.5; p = 0.009), during matches than during training (82% versus 28%; p = 0.003) and during the return leg than the first leg of the championship (58.5% versus 41.5%; p = 0.02).

**Conclusion:**

the frequency of injuries suffered by footballers during the 2019-2020 season was high. These injuries were mostly muscular and tendinous, and the lower limbs were mostly affected. They were associated with physical contact, matches and the return phase of the championship. To be effective, preventive measures should take these characteristics into account.

## Introduction

Le football est le sport le plus pratiqué au monde avec environ 300 millions de joueurs réguliers, enregistrés par la Fédération Internationale de Football Association (FIFA) dans plus de 200 pays du monde [1]. Il est considéré comme un moyen de développement personnel et socio-économique [2], puis de promotion de santé [3]. Toutefois, la pratique d'un sport de contact vigoureux comme le football, caractérisé par la réalisation de diverses activités motrices d'intensité élevée à maximale est associé à un risque supérieur de blessure [4].

Le risque moyen de subir une blessure est 1000 fois plus élevé au football moderne que dans des professions industrielles les plus à risque [5]. Les blessures constituent un évènement indésirable majeur dans la carrière du footballeur. En fonction de sa gravité, la blessure peut avoir des répercussions néfastes sur la performance, la santé voire la carrière réussie du footballeur [6]. Elle peut affecter également les résultats des équipes en ce sens que les équipes les plus performantes sont celles dont les joueurs subissent moins de blessures durant les compétitions [7].

La prévention des blessures est l'une des préoccupations de la FIFA qui a développé des programmes efficaces comme le «11» et la «FIFA 11+» [1]. Elle a suscité également l'intérêt scientifique avec des études épidémiologiques et cliniques. En Europe, une étude longitudinale sur 11 ans a observé que l'incidence des blessures varie de 6,2 à 13,2 pour 1000 h chez les footballeurs [1]. L'entorse et les lésions musculaires ont été rapportées comme étant les traumatismes les plus courants [8]. Les membres pelviens représentent la partie la plus touchée et le genou est la zone la plus affectée par les blessures les plus graves [6]. L'âge, le sexe, le niveau de compétition, le poste de jeu, la surface de jeu et le moment de la saison sont des facteurs associés [3].

En Afrique du sud, l'incidence des blessures des footballeurs professionnels est de l'ordre de 13,4 pour 1000 h, puis la cuisse et la cheville représentent les parties les plus touchées [9]. En moyenne, 3,7 blessures sont enregistrées par match chez les joueurs masculins et féminins au Nigéria [2]. Les joueurs masculins présentent un risque plus élevé, les membres inférieurs sont la partie la plus touchée quel que soit le sexe, la contusion de la jambe est plus élevée chez les hommes et l'entorse du genou chez les femmes [2]. Au Rwanda, la moitié des blessures enregistrées sur trois saisons chez les joueuses était des blessures récurrentes avec la cheville comme la zone la plus blessée [10]. L'âge, le poste de jeu, le niveau de compétition, les blessures antérieures, la qualité de l'équipement de protection, l'amplitude excessive des mouvements de la cheville, les symptômes prémenstruels, l'utilisation de pilules contraceptives sont des associés aux blessures chez ces joueuses [10].

Au Burkina Faso, il y a peu de travaux relatifs aux paramètres épidémiologiques et cliniques des blessures chez des footballeurs, ce qui peut constituer une préoccupation majeure. En effet, le Burkina Faso compte parmi les pays aux ressources limitées en médecine du sport pour une prise en charge appropriée des blessures sportives et la majorité des jeunes pratiquent le football dans l'espoir d'atteindre le plus haut niveau. C'est pourquoi, il nous a paru nécessaire de mettre en œuvre cette étude. Elle vise à déterminer les caractéristiques des blessures subies par des joueurs lors du championnat de football d'élite de la saison sportive 2019-2020.

## Méthodes

**Protocole:** il s'est agi d'une étude transversale, réalisée avec des joueurs masculins de la Ligue du Centre de Football (LCF) à Ouagadougou ayant participé au championnat de football de première division du Burkina Faso. Elle a été réalisée sur une saison sportive 2019-2020 à savoir d'octobre 2019 à juin 2020. Un questionnaire auto-administré a été utilisé pour collecter de données relatives aux caractéristiques sociodémographiques et aux blessures subies par ces joueurs au cours de ladite saison. Un mois avant l'administration des questionnaires, une correspondance décrivant la motivation et le déroulement de l'enquête a été adressée aux clubs, afin d'acquérir une meilleure adhésion à l'étude. L'anonymat et la confidentialité des données collectées ont été strictement respectés conformément à la déclaration d'Elsinki [11].

**Participants:** l'étude a été réalisée avec un effectif de 160 joueurs masculins. Il s'agissait de 16 footballeurs choisis de façon aléatoire dans chacun des 10 clubs de la LCF, retenus par la Fédération Burkinabè de Football (FBF) pour la participation au championnat national de football de première division.

**Collecte des données:** les 160 joueurs retenus ont renseigné une fiche questionnaire auto-administré anonyme 72 heures avant la dernière journée de la compétition. Les fiches questionnaires ont été renseignées sur les terrains d'entraînement, à la fin des séances. Il s'agissait d'une fiche questionnaire utilisée dans des études antérieures et adaptée [10,12]. Elle comportait des questions en relatives aux caractéristiques sociodémographiques et aux blessures.

La blessure a été définie comme un dommage physique survenu pendant des activités régulières de football reconnue par les clubs c'est-à-dire un match officiel ou une séance d'entraînement planifié, à la suite duquel le joueur est incapable de participer pleinement à au moins un match ou une séance d'entraînement [13]. Le joueur a été considéré comme blessé pendant toute la durée du traitement jusqu'à l'obtention de l'autorisation du médecin d'équipe pour participer pleinement aux matchs ou aux séances d'entraînement [13].

**Variables:** les variables indépendantes étudiées étaient les mécanismes de blessures, l'activité sportive au cours de laquelle la blessure est survenue et le moment de la compétition durant lequel la blessure a été subie. Elles ont été rendues opérationnelles en deux modalités: avec contact physique et sans contact physique pour les mécanismes de blessure, durant un match et durant un entraînement pour l'activité sportive au cours de laquelle la blessure est survenue, puis pendant la phase allée du championnat et la phase retour du championnat pour le moment de la compétition durant lequel la blessure a été subie.

Les variables dépendantes étaient les suivantes. (i) le type de blessures: la contusion, l'élongation, le claquage, la déchirure musculaire, l'entorse, la luxation, la fracture osseuse. (ii) la localisation anatomique de la blessure: le pied, la jambe, le genou, la cuisse, la hanche, le dos, l'abdomen, les organes génitaux, le thorax, le cou, la tête, l'épaule, le bras, l'avant-bras, le coude, le poignet et la main. (iii) la gravité de la blessure: la blessure mineure, la blessure légère, la blessure modérée et la blessure sévère. La blessure a été considérée comme mineure, légère, modérée et sévère lorsqu'elle avait nécessité un arrêt de la pratique sportive respectivement d'au plus trois jours, de quatre à sept jours, de huit à 28 jours et plus de 28 jours [14].

**Analyse statistique:** les données collectées ont été traitées avec le logiciel SPSS (IBM Statistics, Version 20). Les statistiques descriptives des variables quantitatives continues ont été présentées sous forme de moyenne (m) ± Ecart type (s). Les fréquences absolues et relatives des variables catégorielles ont été calculées. Le test de chi carré a été utilisé pour déterminer les facteurs associés. Le niveau de signification des tests statistiques a été fixé à p < 0,05.

## Résultats

Caractéristiques sociodémographiques des joueurs enquêtés: l'âge des joueurs enquêtés variait de 17 à 37 ans, avec une moyenne de 26,07 ± 3,01 ans. Les joueurs étaient composés de défenseurs (32,7%), de milieux de terrain (35,6%), d'attaquants (28,9%) et de gardiens de but (2,8%). En moyenne, 24,5 ± 7,09 matchs sur 30 ont été disputés par les joueurs participants soit un taux de 80% des matchs prévus.

**Figure 1:**
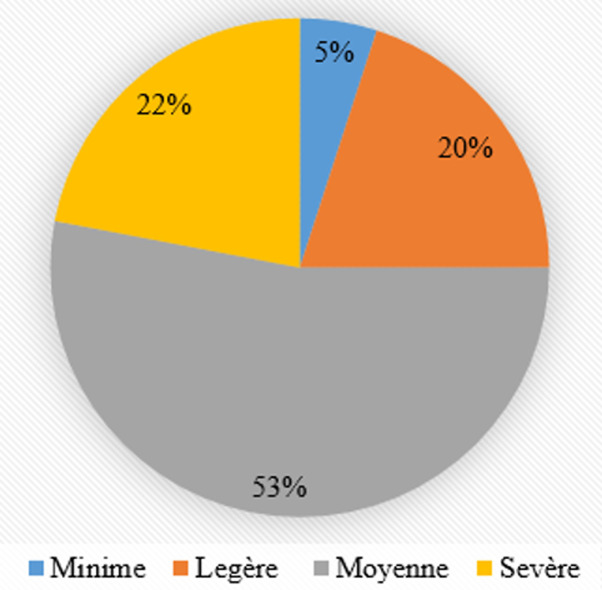
fréquence des blessures observées selon le niveau de gravité chez des joueurs de la Ligue du Centre de Football lors du championnat d'élite de la saison sportive 2018-2019 du Burkina Faso

Fréquence et gravité des blessures: au total, 157 blessures ont été subies par 143 joueurs (89,4%) au cours de la saison. La majorité des blessures (53%) était de gravité moyenne c'est-à-dire qu'elles avaient nécessité huit à 28 jours de récupération (Figure 1).

**Figure 2:**
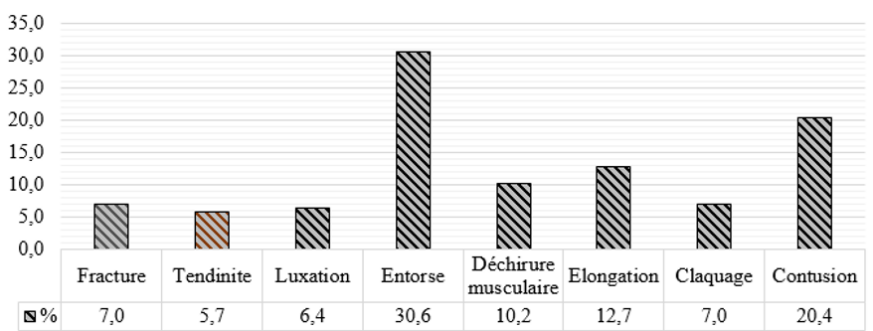
fréquence des blessures enregistrées selon le type chez des joueurs de la Ligue du Centre de Football au cours du championnat d'élite de la saison sportive 2018-2019 au Burkina Faso

Typologie des blessures: l'entorse, la contusion et l'élongation étaient les blessures courantes avec respectivement une fréquence de 30,6%, de 20,4% et de 13,7% (Figure 2).

**Figure 3:**
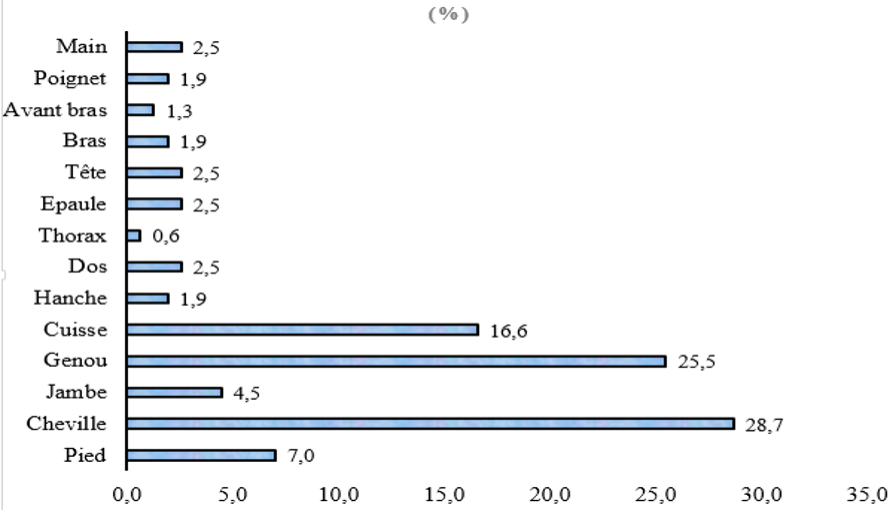
proportion des blessures selon la localisation anatomique chez des joueurs de la Ligue du Centre de Football lors du championnat d'élite de la saison 2018-2019 au Burkina Faso

Localisation anatomique des blessures: la cheville (28,7%), le genou (25,5%) et la cuisse (16,6%) étaient les parties du corps les plus touchées (Figure 3).

Facteurs associés aux blessures: la fréquence des blessures (Tableau 1) était plus élevée après un contact que sans contact physique avec un autre joueur (69,5% versus 30,5; p = 0,009), durant les matchs que pendant l'entraînement (82% versus 28%; p = 0,003) et pendant la phase retour que la phase allée du championnat (58,5% versus 41,5% ; p = 0,02).

**Tableau 1:** facteurs associés aux blessures subies par les footballeurs du championnat d'élite du Burkina Faso au cours de la saison 2018-2019

	Caractéristiques des arbitres		χ^2^	p
**Mécanisme de blessures**	Avec contact	Sans contact		
**Effectifs (%)**	109(69,5)	48(30,5)	0,88	0,009
**Activités sportives**	Entraînement	Match		
**Effectifs (%)**	44(28)	113(82)	0,12	0,003
**Moment de la compétition**	Phase allée	Phase retour		
**Effectifs (%)**	65(41,5)	92(58,5),	1,76	0,02

## Discussion

L'étude avait pour but de déterminer les caractéristiques des blessures subies par des footballeurs au cours du championnat national de première division de la saison sportive 2019-2020 au Burkina Faso. Après les travaux de terrain, il est observé que les joueurs participants étaient relativement jeunes avec un âge de 26,07 ± 3,01 ans. Ils ont disputé un nombre moyen de 24,5 ± 7,09 matchs au cours de la saison, ce qui est relativement élevé quand l'on sait qu'au total 30 matchs sont joués par équipe durant le championnat. Le nombre de blessures subies durant la saison était élevé et touchait la grande majorité des joueurs. L'entorse, la contusion et l'élongation étaient fréquents. La cheville, le genou et la cuisse étaient couramment touchés. Plus de la moitié des blessures était de gravité moyenne. Les blessures étaient plus subies après un contact physique, durant les matchs et lors la phase retour du championnat.

Fréquence des blessures: l'étude a montré qu'un nombre élevé de blessures ont été subies par la grande majorité des joueurs enquêtés. Cela pourrait résulter des exigences psychologiques, physiologiques et physiques élevées qu'impose le football qui compte parmi les sports vigoureux [15]. Les enjeux qu'impliquent certains matchs comme les rivalités historiques entre des équipes, la course à une meilleure place au classement, la lutte contre la relégation en division inférieure, le désir des joueurs de se vendre par ses performances sont également des facteurs qui peuvent accroitre le niveau d'engagement et d'agressivité des joueurs, les exposant ainsi à des blessures [16]. Le contexte du football au Burkina Faso, caractérisé par l'utilisation d'équipements usés et de surfaces de jeu dépourvues de gazons augmentent également le risque de blessures [10,13]. Bien qu'il faille faire preuve de prudence dans la comparaison, cette prévalence semble similaire à celle rapportée par une antérieure [6].

Typologie des blessures: l'entorse et la contusion étaient courantes chez les joueurs. Cela pourrait s'expliquer par le fait que ces lésions concernent le muscle et le ligament qui sont les organes impliqués de façon intense dans la réalisation des activités motrices. Ces organes sont fortement sollicités au football notamment lors de contacts physiques, d'accélérations, de décélérations, d'impulsions, de réceptions après un saut, réalisés sur des terrains inadaptés pourrait en partie expliquer ces résultats [4]. Ce résultat corrobore les résultats des études antérieures [13,17]. Chez les footballeurs semi-professionnels nigérians, la contusion représentait les 3/5 des blessures subies [2]. Ce résultat corrobore les résultats d'une étude précédente réalisée avec les footballeurs kosovars [4].

Localisation anatomique des blessures: la cheville, le genou et la cuisse des joueurs étaient fréquemment victimes de blessure. Ce résultat pourrait s'expliquer par la réalisation des activités motrices intensives comme les sauts, les réceptions des sauts et les changements brusques de directions en réponse au mouvement du ballon ou de l'adversaire qui sollicitent fortement le genou et la cheville [4,6]. La laxité articulaire, prédicteur significatif des blessures et la non utilisation d'équipement de soutien aux articulaires exposerait aussi ces deux articulations intensément sollicitées [18]. La cheville vient en première position comme la zone la plus touchée. Cela n'est pas surprenant quand on sait qu'elle est la principale zone de contact au football, ce qui la rend plus vulnérable [19]. Ce résultat corrobore ceux de plusieurs études précédentes [2,4,6].

Gravité des blessures: plus de la moitié des blessures subies étaient de gravité modérée c'est-à-dire qu'elles ont nécessité huit à 28 jours de récupération. Le manque de personnel médical qualifié en médecine du sport dans les clubs burkinabè peut expliquer ce nombre important de blessures de gravité moyenne. Elle constitue sans doute un obstacle en ce qui concerne le diagnostic précoce et la prise en charge appropriée, ce qui est un frein à la récupération rapides de certaines blessures [1]. La fréquence importante des blessures de gravité moyenne corrobore les résultats des footballeurs au Kosovo [4].

Facteurs associés aux blessures: la fréquence des blessures était plus élevée après un contact physique avec un joueur, puis pendant un match. Cela peut résulter du fait que les activités motrices susceptibles de provoquer une blessure à savoir les tacles, les coups de pieds, les sauts, les réceptions des sauts, les chutes et les pertes de l'équilibre sont beaucoup effectuées pendant les matchs avec une intensité plus élevée [20]. Ces résultats concordent avec ceux des études précédentes réalisées avec des footballeurs de différents niveaux et catégories [13,15,21]. Le taux de blessures était également élevé durant la phase retour du championnat. La phase retour du championnat est généralement caractérisée par une intensification de la compétition avec de multiples enjeux comme la recherche du meilleur rang dans le classement final à savoir le titre de champion ou le maintien dans la division [18]. L'introduction de la Coupe du Faso (Coupe national) à cette période de compétition augmente la charge de travail des joueurs des équipes en compétition avec deux matchs par semaines. La fatigue physique et psychologique résultant des effets cumulés des charges physiques, physiologiques et psychologiques expliqueraient en partie le nombre élevé de blessures durant cette période de la compétition [21].

Limites de l'étude: cette étude est l'une des rares consacrées à l'exploration de la spécificité des blessures chez des footballeurs au Burkina Faso. La principale limite de l'étude est relative à la définition d'une blessure qui varie suivant les auteurs, ce qui implique qu'une certaine réserve soit observée dans les comparaisons. Le fait que l'étude ait été réalisée dans la Ligue du Centre de Football à Ouagadougou constitue une seconde limite en ce sens qu'il n'autorise pas la généralisation des résultats.

## Conclusion

Les résultats indiquent que le nombre de blessures enregistrées chez des footballeurs au cours de la saison sportive 2019-2020 était élevé. Ces blessures étaient majoritairement de gravité modérée avec une fréquence élevée de lésions musculaires et ligamentaires. Elles affectaient les membres pelviens notamment la cheville, le genou et la cuisse. Elles étaient associées aux contacts physiques, aux matchs et à la phase retour du championnat. Ces caractéristiques devraient être exploitées pour l'élaboration d'une stratégie de prévention.

### Etat des connaissances sur le sujet

- *Le football est un sport de contact vigoureux associé à un risque élevé de blessure;*
- *La connaissance des caractéristiques des blessures est indispensable à la définition d'une stratégie de prévention efficace*.

### Contribution de notre étude à la connaissance

- *Les caractéristiques cliniques et épidémiologiques de blessures enregistrées chez des footballeurs burkinabè au cours la saison sportive 2019-2020 corroborent ceux de la littérature;*
- *Ces blessures sont associées aux contacts physiques, aux matchs et à la phase retour du championnat*.

## Remerciements

Aux joueurs, équipes médicales et dirigeants des clubs de la Ligue du Centre de football (Ouagadougou) pour leur contribution à la réalisation de l'étude.
